# Towards Clinical Application: Calcium Waves for In Vitro Qualitative Assessment of Propagated Primary Human Corneal Endothelial Cells

**DOI:** 10.3390/cells13232012

**Published:** 2024-12-05

**Authors:** Xiao Yu Ng, Gary Peh, Fernando Morales-Wong, Rami Gabriel, Poh Loong Soong, Kun-Han Lin, Jodhbir S. Mehta

**Affiliations:** 1 Tissue Engineering and Cell Therapy Group, Singapore Eye Research Institute, Singapore 169856, Singapore; ng.xiao.yu@seri.com.sg; 2 Eye-Academic Clinical Program (ACP), Duke-National University of Singapore (NUS) Graduate Medical School, Singapore 169857, Singapore; 3 Department of Ophthalmology, University Hospital and Faculty of Medicine, Autonomous University of Nuevo León (UANL), Monterrey 64460, Mexico; fernandomoraleswong@gmail.com; 4 Department of Ophthalmology, Duke University Health Center, Durham, NC 27705, USA; rami.gabriel@duke.edu; 5 Ternion Biosciences, Singapore 574329, Singapore; plsoong@gmail.com (P.L.S.);; 6 Corneal and External Diseases Department, Singapore National Eye Centre, Singapore 168751, Singapore

**Keywords:** cornea, corneal endothelium, calcium wave, corneal endothelial cells, cell therapy

## Abstract

Corneal endothelium cells (CECs) regulate corneal hydration between the leaky barrier of the corneal endothelium and the ionic pumps on the surface of CECs. As CECs do not regenerate, loss of CECs leads to poor vision and corneal blindness. Corneal transplant is the only treatment option; however, there is a severe shortage of donor corneas globally. Cell therapy using propagated primary human CECs is an alternative approach to corneal transplantations, and proof of functionality is crucial for validating such CECs. Expression markers like Na-K-ATPase and ZO-1 are typical but not specific to CECs. Assessing the barrier function of the expanded CECs via electrical resistance (i.e., TEER and Ussing’s chamber) involves difficult techniques and is thus impractical for clinical application. Calcium has been demonstrated to affect the paracellular permeability of the corneal endothelium. Its absence alters morphology and disrupts apical junctions in bovine CECs, underscoring its importance. Calcium signaling patterns such as calcium waves affect the rate of wound healing in bovine CECs. Therefore, observing calcium waves in expanded CECs could provide valuable insights into their health and functional integrity. Mechanical or chemical stimulations, combined with Ca^2+^-sensitive fluorescent dyes and time-lapse imaging, can be used to visualize these waves, which could potentially be used to qualify expanded CECs.

## 1. The Corneal Endothelium

**Figure 1:**
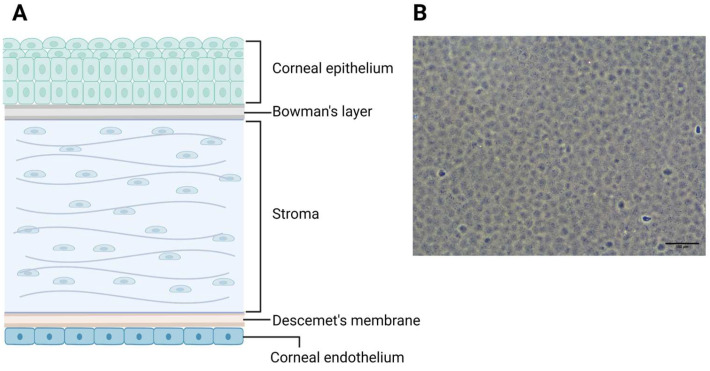
(**A**) Schematic showing the structure of the cornea with 3 cellular layers and 2 acellular layers of basement membrane. (**B**) A figure of a confluent monolayer of corneal endothelial cells in culture. Schematic created with BioRender.com.

The cornea consists of three cellular layers, working in unison to sustain cornea deturgescence, maintaining corneal clarity [1]. The outer-most layer is the corneal epithelium, which, alongside the ocular surface and tear film, serves as the main protective barrier of the cornea [2]. The middle and thickest layer of the cornea—the corneal stroma—is constituted of densely packed interlaced connective tissues formed from a unique arrangement of evenly spaced collagen fibrils wherein the corneal keratocytes reside (Figure 1A) [3,4]. The inner-most layer of the cornea, and arguably the most crucial for maintaining corneal transparency, is the corneal endothelium (CE). It is a monolayer of hexagonal cells (Figure 1B) responsible for the dynamic regulation of corneal hydration between the leaky barrier of the corneal endothelium and the ionic pumps on the surface of corneal endothelial cells (CECs) [5].

The CECs are unable to actively regenerate within the eye as they are growth-arrested at the G1 phase of the cell cycle [6]. It has been estimated that the natural attrition of the central corneal endothelial cells decreases at a rate of approximately 0.6% yearly [7]. There is a significant reduction noted from birth until about 5 years of age. After this period, the rate of decrease in cell density slows down [8,9]. In certain situations, such as genetic diseases (for example, Fuchs’ endothelial corneal dystrophy), this rate can increase to 0.8–3% yearly [6,7]. Physical trauma to the cornea due to injury or intraocular surgical procedures [1,8] has also been shown to increase loss of CECs, resulting in reduced corneal endothelial function and affecting their capacity to maintain corneal hydration. As such, if left untreated, the ensuing edema will result in reduced corneal clarity, leading to low vision and, eventually, corneal blindness [9,10].

Currently, several surgical options are available. Full-thickness penetrating keratoplasty (PK) is the replacement of the full-thickness cornea with donor tissue [11]. However, the disadvantages of PK are its unpredictable astigmatism affecting post-operative vision as well as higher risks of graft rejection compared with newer keratoplasty procedures [12,13]. The rates of full-thickness graft rejection have been reported to be around 7.1% at 3 years and 14.1% at 5 years [14,15]. In the recent two decades, with the advancement in keratoplasty techniques, it is possible to replace only the affected part of the cornea with different variants of deep anterior lamellar keratectomy (DALK) and endothelial keratoplasty (EK), such as Descemet stripping endothelial keratoplasty (DSEK) [16,17], and Descemet membrane endothelial keratoplasty (DMEK) [18,19]. These partial corneal transplants have resulted in improved surgical outcomes, with faster recovery of vision and lower rates of graft rejection (5–9% for DSEK and 0.2–1.7% for DMEK at 5 years) [15,20,21,22].

However, there is a severe shortage of donor tissue available for transplantation globally [23]. This is due to a myriad of cumulative factors including the lack of adequately trained personnel and/or the availability of proper infrastructures; and in some regions, restrictive cultural beliefs [23]. More importantly, amidst the shrinking pool of potential donors as the global population ages, the demand for corneal transplantation gradually increases [5,24]. To circumvent these issues, there has been a push for innovation and research to meet the increased demand for corneal transplantation. With the ability to propagate and expand primary human CECs [25,26,27], and the development of cell-based therapies targeted at the delivery of cultivated CECs either via tissue-engineered endothelial keratoplasty [28] or direct cell injection [29,30], pre-clinical findings have shown that these cellular therapies hold great promise. In fact, a clinical trial from Japan (UMIN000012534) injected expanded primary human CECs supplemented with a rho-kinase inhibitor (ROCK), Y-27632, into 11 patients with bullous keratopathy and showed promising outcomes [31,32]. Recent innovation in cellular therapy incorporating the use of magnetic nanoparticles via cell injection has reportedly enabled more precise delivery of injected cells using a magnetic field and is able to reduce the time required in a face-down position (30 min vs. 3 h) in rabbits compared to conventional cell injection [28,33]. A clinical trial (ClinicalTrials.gov: NCT04191629) sponsored by Emmecell to evaluate the efficacy of a magnetic-nanoparticle cell delivery platform in patients with corneal edema is currently ongoing [34].

With the rapid advancement of cellular therapy comes the demand for the development of robust quality control measures. With the propagation of primary CECs, the proof of cellular functionality is crucial in the validation of the expanded cells. Basic characterization can be performed in vitro, probing for the expression of function-associated pump marker Na-K-ATPase [35] and junctional marker ZO-1 [10], or cell-surface markers such as TAG-2A12 (sPRDX-6) and TAG-1A3 (CD-166) [36]. Ultimately, the expanded primary cells must also be shown to be functional as a cellular monolayer prior to clinical translation, and the “gold standard” proof of functionality is their capacity to reverse corneal blindness within a suitable animal model [37]. Indeed, this has been shown in both rabbits and non-human primates [29,38,39]. However, animal models can be technically challenging, exorbitantly expensive [40], and time consuming. For example, the rabbit’s corneal endothelium is known to regenerate in vivo [41]. As such, to use the rabbit as a model of corneal endothelial dysfunction, the rabbit’s corneal endothelium must be properly and completely removed whilst keeping the Descemet membrane (DM) intact, prior to any procedure to prevent potential repopulation of the rabbit’s naïve corneal endothelium. For non-human primates, though its corneal endothelium is not known to regenerate [42,43], the cost involved is prohibitively expensive [44,45]. In addition, there are both moral and ethical concerns about the use of animals for research [44,46], especially with the rise of companion animals and the status of pets likened to family [47,48]. Therefore, it will be of great benefit, with this aim in mind, that a robust in vitro assay can be utilized for the qualitative assessment of cultured primary CECs; and more importantly, to be able to associate and delineate the functionality of the propagated CECs based on the outcome of such in vitro assay.

## 2. Qualifying CECs: TEER, Ussing Chamber and Patch-Clamp Technique

To infer functionality of a monolayer of CECs within an in vitro setting, it is possible to demonstrate active metabolic ion transport [49], where the permeability observed serves as a key indicator of barrier integrity. The most used methods are trans-endothelial electrical resistance (TEER) measurements [49,50] and Ussing chamber measurements [49,51,52,53,54]. TEER provides a physiological index that reflects the transport rate of ions through the paraendothelial pathway for the measurement of cellular permeability, where higher TEER values indicate stronger junctions between cells [55,56]. Measurements of TEER have been widely accepted as a quantitative approach to study barrier function and tight junction dynamics [57,58]. For example, Bartakova et al. demonstrated that human CECs with a well-organized hexagonal shape exhibit higher TEER values compared to those with a fibroblastic morphology, highlighting the link between cell morphology and barrier integrity [59].

### 2.1. TEER: Ohm’s Law Method

Electrical resistance (measured in ohms) is a quantitative measure of the barrier integrity of a cell layer [55,60]. In the classical setup for the measurement of TEER, an alternating current (AC) signal is applied across a cell layer grown on a semipermeable membrane with the electrodes placed on both sides of the cell layer. Both the current and voltage across the cell layer are measured [61], and Ohm’s Law is used to calculate and determine the resistance [61]. Several commercial systems run on this configuration; EVOM2 (World Precision Instruments, Sarasota, FL, USA) [62] and Millicell ERS (EMD Millipore, Darmstadt, Germany).

### 2.2. TEER: Impedance Spectroscopy

TEER can also be measured via impedance spectroscopy, where the frequency of an applied AC voltage is swept while measuring the amplitude and phase of the resulting AC current [55,60]. Impedance spectroscopy is a more complex setup that requires more advanced equipment such as a lock-in amplifier and function generator [61,63]. The ECIS (Applied BioPhysics, Troy, NY, USA) [50,57] and cellZscope^®^ (nanoAnalytics, Munster, Germany) are commercial devices that provide impedance spectroscopy measurements.

TEER is widely adopted as it is non-invasive and can be used to monitor live cells [55]. Although powerful, TEER measurements can be subjected to variability [60,61], due to the highly sensitive nature of the methodology. Specifically, careful handling of electrode placement as well as manipulation is critical for avoiding disrupting the cellular layer and compromising results [61]. Uniformity of the current density generated by the electrodes across the cell layer also has a significant effect on TEER measurements [55,58,60,64]. If the current distribution across the membrane is not uniform, the effective area of the membrane is reduced, leading to an overestimation of the TEER value and the resistance of the cellular barrier [58,60,61,64]. Overall, measuring TEER requires careful planning and execution, which renders itself tedious as a quality assurance test for cell therapy.

### 2.3. Ussing Chamber

The Ussing chamber consists of a cylindrical tissue holder, to which potassium chloride (KCl)-filled glass columns, agar bridges, and calomel electrodes are attached [65,66]. The tissue is mounted and compressed between the two chamber halves [66,67], with the apical side facing one half and the basolateral side facing the other, effectively separating the solutions bathing each side [67,68]. The enclosed baths on the apical and basolateral sides are perfused via a glass circulation reservoir mounted above the chamber [66,67]. This reservoir is water-jacketed to maintain the superfusate (i.e., CO_2_/HCO_3_−-buffered Ringer solution, such as Krebs bicarbonate Ringer (KBR)) at 37 °C, using a gas-lift mechanism to drive circulation and maintain gas tension (95% O_2_ and 5% CO_2_) [67,68]. Such a configuration allows researchers to precisely control the chemical and electrical conditions on either side of the membrane.

The Ussing chamber has been used to quantitatively validate the corneal endothelium’s “pump” function [53,65,69,70] through the measurement of electrical potential across an intact corneal endothelium, providing insights into the cell layer’s pump activity [69]. However, implementing the Ussing chamber is complex and requires careful attention to numerous experimental details. Before starting an experiment with the Ussing chamber, it is essential to eliminate any electrical bias within the measurements [67]. Bubbles, which can potentially interfere with the chamber circulation, must be removed, and all leaks in the system must be secured [67]. The “zeroing” process to eliminate electrical bias can only be performed when the superfusate reaches 37 °C [67]. Electrode resistance may vary over time, causing asymmetries; hence, equilibration of the electrodes should be performed to offset the voltages generated before the start of each experiment [65]. It is important to ensure both sides of the reservoir are level to maintain equal hydrostatic pressure [65,67], as any imbalance in height of the solution or gas lift rates [67] can cause the tissue to balloon into one chamber, potentially bending and damaging it [65,67]. Additionally, when the two half-chambers are pressure clamped together, edge damage to the outer circumference of the tissue can occur [67,71,72], introducing error in the total resistance measurement. The error can be more significant with smaller exposed tissue areas due to the higher edge-to-surface-area ratio [71,72]. The complex setup also makes it challenging to clean, potentially increasing the risk of contamination [67]. Finally, Ussing chamber setups are highly sensitive to external factors such as the distance between the electrode and measurement membrane, pH changes, temperature fluctuations, cell shedding, and vibration sensitivity of the instrumentation [49]. These technical challenges make it unsuitable for use in a cell therapy setting. Furthermore, the generated tissue-engineered corneal endothelial grafts are delicate and cannot risk any form of damage. While expanded CECs can be tested using inserts in the Ussing chamber, mastering this technique for routine quality assurance remains a significant hurdle.

### 2.4. Patch-Clamp Technique

The patch clamp method is an electrophysiological technique that uses a microelectrode (patch pipette) to form a tight seal on the cell membrane. The small patch of membrane can be ruptured by applying positive pressure or includes pore-forming agents in the patch pipette that create small perforations in the membrane, allowing electrical access to the whole cell [73,74]. There are two modes of patch clamping, namely, the voltage clamp mode and the current clamp mode. For voltage clamp mode, a fixed voltage is applied and the amount of current passing through the cell membrane is measured. This mode measures the activity of voltage-dependent ion channels via changes to the membrane potential, where the permeability of the membrane to different ions can be determined [74].

Alternatively, for current clamp mode, a fixed amount of current is injected into the cell, and the resulting change in membrane potential is measured [74]. To accurately acquire the resting membrane potential (RMP) of the cell, it should be recorded immediately once the cell membrane is ruptured or in perforated mode without any current injection. The RMP can be used to infer the health of the cell, providing a means to assess the quality of expanded CECs in vitro [75,76]. Liu et al. employed this method to determine the quality of cultured rabbit CECs tissue-engineered onto a porcine DM, by comparing their membrane potential to the freshly isolated rabbit corneal endothelium [75]. The action potential amplitudes of both groups are over −80 mV, thereby inferring functionality of their rabbit CECs on porcine DM tissue-engineered graft [75].

Patch clamp recordings typically require a well-calibrated setup. Briefly, cells seeded on glass coverslips are placed in a specialized recording chamber, equipped with controlled inflow and outflow of extracellular solution and a facility to attach the grounding electrode [74]. The recording chamber is fixed onto the stage of a microscope, preferably with a camera attached and connected to a computer, to facilitate the positioning of the patch pipette onto the cells [74]. Micromanipulators (hydraulic, piezoelectric-controlled or motorized) that are low-noise provide fine control movement that are mounted on a separate platform to move the patch pipette into position [74].

The patch-clamp technique is very sensitive to environmental noise, and excessive noise can completely drown out the electrophysiological signal. In addition, any vibrations may affect the readout (i.e., dielectric noise, thermal noise, power supplies, fluorescent lights, etc.) [77]. Therefore, the whole setup must be placed on an antivibration table surrounded by a Faraday cage to shield from electrical interference [74]. Ideally, the system should be isolated from the main laboratory space to avoid extraneous noise. A well-trained scientist or technician is required to perform recordings, making it a delicate experiment to conduct. Consequently, implementing it as a routine quality control measure in corneal endothelial cell therapy may be challenging.

## 3. Calcium’s Role in CECs

Calcium is crucial for many cellular processes. Increases in cytoplasmic calcium regulate various cellular functions [78,79], such as muscle contractions [78,80,81] and neurotransmitter [78,82] release in excitable cells, as well as transcription during the cell cycle or apoptosis [78,83] in non-excitable cells. Calcium signals can be characterized by distinct spatiotemporal dynamics and can be interpreted by calcium-sensing effectors to elicit specific cellular responses [78,79,84,85,86,87].

Numerous studies on the spatiotemporal dynamics of calcium signals have been conducted within in vitro cell culture [78], including CECs. Calcium in corneal irrigating solution is essential for maintaining intercellular junctional complexes and the integrity of the barrier function of CECs [88,89,90]. Studies using bovine CECs showed that in the absence of extracellular calcium, bovine CECs appeared rounded with a “cobblestone” morphology and exhibited apical junction breakdown when observed under scanning electron (SEM) and transmission electron microscopy (TEM) [91,92,93]. Ramachandran et al. reported that calcium depletion led to the disassembly of adherens junctions (AJs), which subsequently accelerated the breakdown of tight junctions (TJs) through an increased contraction of the peri-junctional actomyosin ring (PAMR) [94,95,96]. PAMR is a thick band of actin cytoskeleton proximal to the apical junction complex of the CE [94,95], which is structurally linked with the AJs and TJs, through proteins like zonula occludens-1 (ZO-1) [94,97,98]. It has been speculated that excessive actomyosin contraction of PAMR reduces cell–cell tether and eventually compromises barrier integrity [94,96,99,100,101].

Bovine CE incubated in calcium-free media was shown to swell rapidly due to increased paracellular permeability [91], but the swelling can be stopped with the addition of calcium. Permeability is crucial for corneal endothelial function, as CE acts as an active ionic pump that maintains corneal fluid balance and prevents swelling and edema of the cornea [35,94,102,103,104,105]. Therefore, calcium is essential to corneal endothelial function [82,83,91,94,106,107].

Calcium signaling patterns at the single-cell level include calcium spikes, intracellular calcium waves (CWs) and more complex oscillatory behaviors [78]. In addition to intracellular CWs, intercellular CWs can occur with increases in calcium concentration that spread across neighboring cells [78], appearing as waves radiating away from an initiating cell [86]. This phenomenon has drawn attention to using calcium waves as a potential tool for qualifying CECs.

## 4. Inter-/Intracellular Calcium Waves: Mechanisms and Implications in Cellular Communication

Calcium waves are a fundamental aspect of cellular signaling, often relying on the interplay between inositol-1,4,5-trisphosphate (IP3) and its receptors (IP3Rs) found on the endoplasmic reticulum (ER) membrane. IP3 binds to IP3Rs to stimulate the release of calcium ion (Ca^2+^) from the ER [78], a principal intracellular calcium store. These IP3Rs are not merely passive channels for Ca^2+^; they are highly sophisticated Ca^2+^-permeable channels whose activity is finely modulated by several factors, including Ca^2+^ itself [86,108,109,110,111]. The relationship between IP3Rs and Ca^2+^ is nuanced, governed by a biphasic calcium concentration dependency [112,113,114,115,116]. This dynamic regulation means that the probability of the IP3R channels opening does not increase linearly with intracellular calcium concentration [Ca^2+^]I [86,108]. Instead, there is an optimal [Ca^2+^]i, around 200 nM, where the open probability of IP3R channels reaches its peak [112]. The binding of IP3, particularly at elevated concentrations, alleviates the calcium inhibition on these channels [86,108]. This intricate balance between IP3 and [Ca^2+^]i is vital for precise control of calcium signaling. It acts as a trigger for the calcium-induced calcium release (CICR) mechanism, where an initial release of calcium sets off a cascading release, effectively amplifying the signal within the cell. This mechanism is integral to the generation and propagation of abrupt and rapid CWs across cells [108,117,118,119]. These waves are sustained by continuous cycles of calcium release, subsequent diffusion, and further rounds of CICR, creating a self-propagating signal, known as intercellular calcium waves, that can traverse considerable distances within tissues [108,120,121].

Intercellular CWs highlight the complexity of cellular communication, enabling a synchronized response throughout extensive networks of cells within tissues and organs. These waves are crucial for a range of essential physiological processes, such as tissue development, wound healing, and the synchronization of the nervous and cardiovascular systems. Central to the propagation of these CWs are gap junctions that are made up of proteins called connexins [86,122]. These proteins assemble into connexons, forming direct connections between cells, enabling unrestricted passage of ions and small signaling molecules across the cellular divides, effectively erasing boundaries [86,123,124,125,126]. Among the various connexin isoforms, Cx43 stands out for its widespread presence [127]. The distinct properties of the connexins that make up each gap junction are critical in determining their physiological roles, such as permeability and gating behaviors [86]. These features enable the passage of small signaling molecules, such as Ca^2+^ or IP3 (less than 1.5 kDa), through the junctions, a process crucial for triggering and adjusting Ca^2+^ release from the intracellular stores of adjacent cells [123,128]. Without gap junctions, intercellular CWs could not be propagated, as reported for glioma cells [129]. Conversely, expressing Cx43 gap junctions in glioma cells not only introduced CW propagation but also revealed a strong correlation between the extent of CW propagation and the expression level of connexin [129]. Moreover, several studies have shown that IP3 can migrate between cells through gap junctions to initiate CWs. This was demonstrated in experiments where caged-IP3 microinjected into glial cells revealed that IP3 diffusion through gap junctions is essential for CW initiation. This finding is further supported by evidence that cells with V84L-mutant Cx26 gap junctions, which are impermeable to IP3, are unable to generate CWs in response to IP3 [86,130]. Together, these observations highlight the significance of gap junctions, not just as structural bridges but as vital passage for signaling molecules involved in directing the intricate interplay of intercellular CWs.

In addition to IP3, paracrine signaling involving the extracellular release of ATP [78,86] has been suggested as a facilitator of intercellular CWs [78]. These waves frequently coincide with ATP bursts [131,132,133], evidenced by elevated ATP concentrations in the culture medium around cells following the start of an intercellular CW. Furthermore, the direct application of ATP to cells can trigger intercellular CWs [134]. Several studies have indicated that various stimuli can trigger non-excitable cells to release ATP [135,136]. These stimulations include mechanical stimulation of cells via exposure to shear stress, cell swelling due to hypotonic conditions, increase in intracellular IP3 levels, and fluctuations of extracellular or intracellular Ca^2+^ concentrations [137]. Once released, ATP traverses the extracellular space and binds to receptors on adjacent cells that predominantly generates intercellular CWs through the activation of G-protein-coupled P2Y receptors [123,138,139,140,141], such as P2Y1, P2Y2, and P2Y4 [142,143,144]. Thus, the spread of intercellular CWs via paracrine signaling is shaped by the rates at which ATP is released and metabolized, as well as by the different types of purinergic receptors expressed.

## 5. Calcium Waves in Corneal Endothelium Cells: Insights and Impact

The corneal endothelium is a single layer of cells lining the inner surface of the cornea and is crucial for regulating corneal hydration, maintaining corneal transparency and optical quality. Primary human CECs have limited capacity to regenerate within the eye [145]. Few studies have examined the relationship between CWs and the well-being of these CECs.

Using bovine CECs, Justet et al. investigated and highlighted the importance of fast calcium waves (FCWs), which are initiated within minutes of a mechanical injury [146]. These FCWs, mediated by IP3 signaling and oscillating in response to ATP, play a crucial role in preventing excessive apoptosis in healing cells [146]. Suppressing FCWs significantly increases apoptosis, underscoring their essential function in preserving cellular integrity post-injury [146]. Further studies by Justet et al. revealed that blocking FCWs leads to a substantial decrease in the proliferation rate of bovine CECs in non-injury tissue restitution models [147], indicating that FCWs are not only protective against apoptosis but are also crucial for endothelial recovery. Extracellular ATP triggers FCWs across various cell types, including bovine CECs [146,147,148,149]. Preincubation with ATP before exposure to cell death inducers significantly limits apoptotic cell increase, with increased [Ca^2+^]i being a key player in the ATP-mediated protective effects [147]. Additionally, Chifflet et al. identified slow calcium waves (SCWs) that developed an hour after wounding, suggesting a layered response to injury where different types of CWs have temporal roles in the healing process [150]. Like FCWs, SCWs facilitate wound healing in bovine CECs, illustrating the complexity of Ca^2+^-mediated signaling in corneal endothelium recovery [150].

Besides wound healing, CWs have also been implicated to correlate aging bovine CECs and those under stressful conditions. Research on extended cultures (defined as 21–30 days) of bovine CECs showed a decrease in CW activity, associated with morphological changes reminiscent of aging or hypoxic conditions in human CECs [151,152]. The decrease in CW propagation, along with increased cell size and surface area, points to a decline in ATP-mediated paracrine intercellular communication (PIC), indicating the sensitivity of CWs to cellular aging and environmental conditions [151]. D’hondt et al. previously demonstrated the effect of increased cell size and cell surface area in bovine CECs cultured over a long period, similar to the effect of aging in human corneas [151]. Long-term culture significantly reduces Ca^2+^ wave propagation; specifically, the percentage of responsive cells decreases with greater distance from the mechanically stimulated cell [151]. Ca^2+^ wave was observed to cover six cell layers when bovine CECs were cultured for a short period (10 days), while it only reached three cell layers in cells cultured for a long period (21 days) [151]. The total area of cells (active area) reached by the Ca^2+^ wave also decreased by ~26% in cells cultured for a long period [151]. Both the active area and the intensity of the Ca^2+^ wave is markedly diminished in the long-term culture, suggesting that this reduction in wave activity could serve as a potential quality indicator for CEC health and functionality.

The combined observations of CWs produced in wound healing and their diminished propagation in ‘aged’ bovine CECs made it plausible to consider utilizing CW as a potential novel biomarker for determining corneal endothelial health. Diminished CW propagation in aged bovine cells suggests that CWs reflect barrier function as well as vitality of the CECs. Further research elucidating the precise relationship between CWs and human CEC health is crucial for definitively establishing CWs as a biomarker. For example, using primary human CECs from young and aged donors can be performed to delineate the sensitivity of CWs to cellular aging as well as cellular senescence. This approach holds promise due to its potential advantages over conventional methods like immunofluorescence as markers for CECs such as Na^+^ K^+^ ATPase, ZO-1, sPRDX-6 and CD-166 provide only broad characterization [36,153]. Electrical impedance measurement, while not specific to CECs, provides a sensitive measurement of electrical resistance displayed by the CECs, which can be inferred to show the integrity of the CEC monolayer and hence the barrier function (i.e., functionality) of the cells. However, as described above, TEER and the Ussing chamber require complex setup, making them impractical for qualifying CECs for clinical applications. Calcium is essential for maintaining intercellular junctional complexes and the integrity of the barrier function of CECs [88,89,90]. Studies conducted on bovine CECs confirmed that in the absence of extracellular calcium, bovine CECs appeared rounded and exhibited apical junction breakdown [91,92,93]. Ca^2+^ wave detection provides an in-depth look at intercellular calcium signaling and successful transmission of Ca^2+^ wave indicates an intact monolayer of CECs, while the active area covered by the Ca^2+^ wave has been demonstrated to decrease while cell size increased (decreasing cell quality) in cultured bovine CECs [151]. Barrier integrity and vitality of the CECs can be inferred from the active area of Ca^2+^ wave propagation. Ca^2+^ wave, though not specific to CEC, is sensitive to the detection of minute cellular changes that affect the overall health and quality of cultured CECs. Lastly, functional validation for each batch of primary human CECs in animal models is time-consuming and costly, and it is thus not ideal either. Overall, measuring CWs could offer an efficient and practical approach for assessing the health of cultured primary human CECs, especially in the context of cell therapy.

## 6. Methods of Inducing and Capturing Calcium Waves in CECs

**Figure 2:**
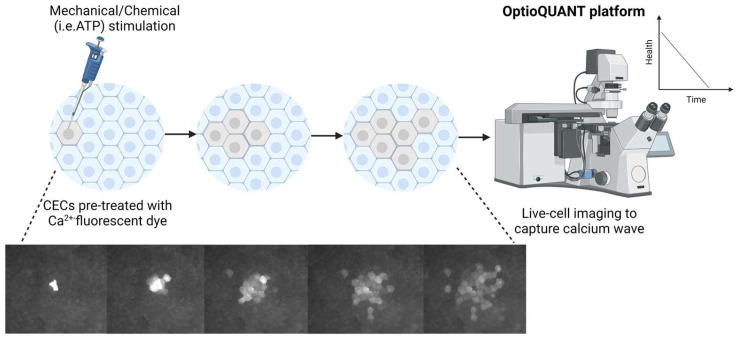
Calcium wave propagation from a single-cell mechanical stimulation captured on OptioQUANT platform to assess and demonstrate intact cell–cell interactions in human CECs. Schematic created with BioRender.com.

Inducing and capturing CWs in primary CECs is a sophisticated process that involves a series of steps to stimulate these cells and then visualize the resulting calcium signals (Figure 2), for example, Ternion’s modular high speed and high-resolution live imaging platform, OptioQUANT [154], to study the physiological and pathological responses of cultured primary human CECs (Figure 2). When stimulating CECs to induce CWs, several established methods may be exploited to mimic natural cellular responses to activate specific pathways.

The most common approach for stimulating cells is through mechanical stimulation. This direct method involves gently contacting a cell or group of cells with a micropipette or mechanical probe [123,137,146,151]. Applying localized pressure or physical pushing triggers a cascade of calcium signaling within cells [123,137,146,151], mimicking the natural responses CECs exhibit under mechanical stresses [123,137,146,151]. The initial touch can lead to a rapid effect, causing CWs to spread to neighboring cells. Mechanical stimulation is also relatively easy to set up, achievable with CECs seeded on a chambered slide. The CECs on a chambered slide are treated with 10 μM Fluo-4 AM in phosphate-buffered saline (PBS) for 30 min at 37 °C [123]. Then, the CECs are washed several times with PBS and rested for 5 min before stimulation is started by briefly touching (~1 s) a single cell with a glass micropipette (tip diameter < 1 μm), coupled with a piezoelectric crystal nano-positioner (0.2 to 1.5 V) [123]. The nano-positioner is operated through an amplifier mounted on a micro-manipulator [123]. Spatial changes in [Ca^2+^]i are measured with the confocal microscope at excitation/emission 488/530 nm, using a long-pass emission filter [123]. The images are analyzed by defining a region of interest (active area) of the total surface area of responsive cells [123].

Chemical stimulation, however, takes a more systemic approach by incorporating distinct chemical agents that are identified for their ability to initiate CWs. ATP is one such chemical agent that can be added to CECs to trigger CWs. Its mechanism involves the activation of purinergic receptors located on the cellular membrane, setting off a chain reaction that triggers the release of Ca^2+^ from the cells’ internal stores [123,138,139,140,141]. The complexity in this technique requires the careful calibration of ATP’s concentration and a delivery system that ensures precise and uniform distribution to cells. The method of chemical application can vary, extending from a bath perfusion, which exposes all cells to the chemical, to a more refined and localized application using a micropipette, targeting specific cells or cell regions for a more controlled response [155,156,157]. The most advanced photon-activated compounds and optogenetic techniques use light as a precise tool for cellular stimulation and enable researchers to precisely control the initiation timing and positioning of Ca^2+^ signals within the cells [158,159].

This approach utilizes focused light beams to activate target compounds, such as caged calcium or caged IP3, thereby initiating signaling waves [158,159]. Such a technique highlights the precision achievable in modern cellular biology and opens new avenues for investigating the complex spatial and temporal dynamics of CWs in living cells. Furthermore, analyzing CWs could potentially evolve into a qualitative proxy for assessing the functionality of propagated cells for cellular therapeutics and serve as a functional criterion used for characterization of CECs for clinical use as diminishing CWs has been associated with cellular aging or unhealthy cells [151,152]. The precision in initiating CWs through a micropipette allows for controlled initiation and quantification of CWs spread across a monolayer of CECs. This can be used to calculate a ratio or index indicative of the evaluated confluent culture of CECs. Since CWs are relatively quick and easy to capture under fluorescence imaging, this method could be readily incorporated into laboratories handling CECs for cellular therapeutics.

To visually observe calcium waves (Figure 2), researchers may choose to label cells with Ca^2+^-sensitive fluorescent dyes, such as Fluo-4 AM [123,151]. These dyes are designed to permeate cell membranes and fluoresce upon binding to Ca^2+^, and are used as an indicator of the change in [Ca^2+^]i. Once the cells are primed and stimulated, they are placed under a fluorescence microscope setup, with a tailored light source emitting specific wavelengths necessary to activate the dye, along with filter sets designed to capture its fluorescent emissions [123,137,146,151]. Such a configuration facilitates the real-time visualization of CWs or their progression through time-lapse imaging [146]. Hence, data acquisition is best commenced prior to the initiation of cellular stimulation and continues beyond it to ensure comprehensive recording of the entire cycle of calcium waves [146].

Yet, the quest to balance high-quality image acquisition through adequate dye concentration with the need to prevent cellular toxicity or disruption of normal functions remains challenging. Optimization together with fine-tuning is essential for effectively recording CWs without harming the cells. Preserving the cells’ physiological state, including consistent temperature, pH, and osmolarity, is just as critical during both the dye loading and stimulation stages. Any shift from their optimal conditions could not only reduce the dye’s effectiveness but could also disrupt cellular responses, potentially influencing the results. Furthermore, the issues of photobleaching and phototoxicity require careful attention, especially under prolonged and intense fluorescence lighting, which can degrade the dye’s luminosity and even damage the cells, altering their reaction to stimuli. Addressing these issues requires careful handling of chemicals and recording techniques, creating the groundwork for a robust methodology in evaluation of cultured primary human CECs.

## 7. Conclusions

As the global demand for corneal tissue needed for corneal transplantation increases, new surgical modalities have been developed to alleviate the situation. Culturing primary human CECs for cell therapy is one way to boost the supply of CECs. While the introduction of in vitro cell culture has become more robust, there comes the need to qualify the cultured primary human CECs to fulfil regulatory requirements and ensure that the in vitro process does not alter the CECs and that they are comparable to the CECs from direct corneal transplants. In addition to staining for CEC markers (CEC-identity), many groups have tried to include indirect methods to qualify the function of these cells such as the measurement of TEER or the use of Ussing chamber. As discussed, these methods are hard to maneuver and are not suitable in a cellular therapeutic setting.

Calcium waves in CECs play a significant role in intercellular communication and have been observed during the wound healing process. These waves exhibit distinct timing in relation to the healing event. Immediately after wounding, a fast calcium wave propagates quickly and then diminishes. Approximately an hour after the initial wounding, a slow calcium wave emerges, associated with the depolarization of the plasma membrane potential of border cells, and contributes to the healing process. The propagation of these waves involves complex interactions with various channels as well as exchangers, including the sodium channel and the sodium–calcium exchanger [150,160]. The modulation of these waves by different factors can affect the rate of wound healing, suggesting that careful observation of calcium signaling in cultured primary human CECs could provide valuable insights into their health and functional integrity. In addition, CW activity was decreased while cell size and cell surface area increased in bovine CECs cultured for an extensive period [151]; since cell morphology and cell size are good indicators of the quality of cultured CECs, such a correlation reveals the potential for CW to be a parameter in the quality control of cultured CECs. Therefore, monitoring calcium wave patterns and responses could potentially be included as a quality control measure, in addition to functional-associated markers, to provide fuller insight into the quality of cultured primary human CECs.

## Data Availability

Data sharing is not applicable to this article as no datasets were generated or analyzed.
